# A Modular IoT Platform for Real-Time Indoor Air Quality Monitoring

**DOI:** 10.3390/s18020581

**Published:** 2018-02-14

**Authors:** Mohieddine Benammar, Abderrazak Abdaoui, Sabbir H.M. Ahmad, Farid Touati, Abdullah Kadri

**Affiliations:** 1 Department of Electrical Engineering, College of Engineering, Qatar University, Doha 2713, Qatar; abderrazak.abdaoui@qu.edu.qa (A.A.); sabbir_ahmad9269@yahoo.com (S.H.M.A.); touatif@qu.edu.qa (F.T.); 2 Qatar Mobility and Innovation Center, QSTP, Doha 210531, Qatar; abdullahk@qmic.com

**Keywords:** indoor air quality monitoring, WSN, Internet-of-Things, Emoncms

## Abstract

The impact of air quality on health and on life comfort is well established. In many societies, vulnerable elderly and young populations spend most of their time indoors. Therefore, indoor air quality monitoring (IAQM) is of great importance to human health. Engineers and researchers are increasingly focusing their efforts on the design of real-time IAQM systems using wireless sensor networks. This paper presents an end-to-end IAQM system enabling measurement of CO_2_, CO, SO_2_, NO_2_, O_3_, Cl_2_, ambient temperature, and relative humidity. In IAQM systems, remote users usually use a local gateway to connect wireless sensor nodes in a given monitoring site to the external world for ubiquitous access of data. In this work, the role of the gateway in processing collected air quality data and its reliable dissemination to end-users through a web-server is emphasized. A mechanism for the backup and the restoration of the collected data in the case of Internet outage is presented. The system is adapted to an open-source Internet-of-Things (IoT) web-server platform, called Emoncms, for live monitoring and long-term storage of the collected IAQM data. A modular IAQM architecture is adopted, which results in a smart scalable system that allows seamless integration of various sensing technologies, wireless sensor networks (WSNs) and smart mobile standards. The paper gives full hardware and software details of the proposed solution. Sample IAQM results collected in various locations are also presented to demonstrate the abilities of the system.

## 1. Introduction

Indoor and outdoor air quality (AQ) is becoming a major concern for the public and policy makers. Recently, Europe and USA have reduced the emissions of several airborne pollutants [1,2] such as carbon monoxide (CO), sulfur dioxide (SO_2_) and lead (Pb). Nitrogen dioxide (NO_2_), ozone (O_3_), particulate matter (PM) and some other organic compounds are also considered as a serious threat to health. In addition, several statistical studies showed that people spend up to 90% of their time in enclosed environments such as offices, schools, homes, and shopping malls [3,4]. For all these reasons, real-time indoor air quality monitoring (IAQM) is required. Collecting data concurrently from various locations and making it available ubiquitously enables interested users to be informed about AQ in their chosen or planned places. Additionally, it is often difficult for air conditioning managers who control ventilation inside buildings to obtain real-time indoor air quality (IAQ) data, and hence resort to overventilation to ensure safe levels of pollutants in their buildings. This overventilation is costly, particularly in harsh environments (very hot or very cold environments), as outside air pumped into the building for diluting pollutants needs energy either for cooling or for heating.

Since IAQM can vary significantly from one location to another even inside the same building, WSN may be used for simultaneous and real-time monitoring of air quality in multiple target locations. Several previous works presented significant contributions in this area, and will be discussed in the present section. Brienza et al. [5] presented a low-cost cooperative monitoring tool that allows a real-time monitoring of the concentration of some polluting gases in various areas of a city. In this solution, the system is confined to outdoor AQM and at each location individual sensor boards are installed to post the data measurements directly through the Internet to a centralized server. The sensor node is based on an embedded solution from Libelium, which is provided with a dedicated integrated development environment (IDE) for composing firmware. The server is implemented using PostgreSQL for data base management, the webserver Apache, Google Maps Application Programming Interfaces (APIs) for sensor visualization and the Java script library for graph visualization. Their system is very interesting since it enables online display of the data measurements. However, the system does not include a local network for the communication between neighboring sensor nodes; each of these nodes posts its collected data directly to the server. In addition, this system is dedicated for users with specific end-user devices.

Lambebo and Haghani [6] implemented a real-time temperature and greenhouse gas concentration monitoring system using WSN. The gateway is an open source low-power hardware based on Arduino Uno Microcontroller and radio module XBee IEEE 802.15.4 for the ZigBee communication. The gateway ensures the relay of data to WiFi network and the acquisition of data measurements from all the local sensor nodes. A server communicates with the local network through the base station with WiFi. At the server, a database and website were developed to store and disseminate the environment data. The gateway simply receives the packets from the local sensor network and relays these through WiFi interface; neither local storage at the gateway nor data resubmission in the case of Internet disconnection are provided.

Fioccola et al. [7] described a system based on Arduino board and a cloud-based platform that manages data obtained from AQ sensors. A comparison between two cloud computing service models and between two IoT communication protocols has been performed. However, the presented system is based on wired technology, which limits the mobility of the sensors and their positioning. In addition, the rescue mechanism, in the case of Internet disconnection at the gateway, has not been addressed.

Tsang et al. [8] designed a ZigBee network for IAQ monitoring system. They introduced an energy saving ZigBee WSN network with low latency and high throughput. However, in their work, the authors have proposed simulation of WSN for IAQM without implementation and deployment of embedded devices. In their work, the authors designed the simulation scenario using OPNET, software dedicated to network problems.

Postolache et al. [9] presented a WiFi-based network for IAQM; their system incorporates sensor data processing based on neural networks for the detection of air pollution events and sensors’ abnormal operation. Each sensor node transmits the data using TCP/IP communication to the main processing and control unit (laptop PC), which performs data logging, data processing, and Web publishing through a LabVIEW web server. Their system is still in laboratory and pending deployment in indoor sites.

Jelicic et al. [10] presented a WSN for IAQM that features people presence sensing and activity recording, and long node autonomy [11]. However, the authors limited their study to simulate the power consumption of a small WSN in an IAQM environment. The data are posted to a web-server, through the coordinator and the router without any local storage or resubmission of the lost data packets.

Kim et al. [12] analyzed the issues, infrastructure, information processing, and challenges of designing and implementing an integrated sensing system for real-time IAQM. The sensor node is based on the MPU MSP430 micro-controller, the RF chip CC2500 and several gas sensors. The sensor node sends data through serial communication interface. The AQ data are stored in a middleware and posted to a cloud platform. The solution is not embedded since the sensing device is directly wired to a computer. Moreover, the solution did not tackle the issue of data reliability when the Internet connection is lost.

Firdhous et al. [13] proposed an IoT-based IAQM system limited to monitoring O_3_ concentrations near photocopy machines. The IoT device communicates with a gateway node over Bluetooth, which in turn communicates with the processing node via WiFi. The system proposed by the authors is limited to the monitoring of ozone produced in offices by the photocopier machine.

Hassan et al. [14] proposed a wireless electronic nose to detect health-endangering gases in indoor environments. However, their work focused on the gas identification using encoding models. Although the approach offers a reduced computational power and memory requirement, the work was limited to some mathematical tools, which were not validated against AQ data from any site.

Chen et al. [15] described a system for monitoring CO_2_ in indoor environments. The main focus was on providing people in these environments with information on CO_2_ levels through their mobile devices, which read quick response (QR) codes.

In this work, we propose an end-to-end solution for IAQM; the system integrates wireless AQ sensor nodes, advanced embedded gateways, and an IoT server. The proposed system stores and posts live data on the server. Additionally, it includes data backup tool at the sensor nodes and gateway levels, ensuring no data loss in the case of loss of communication within the system. The main contribution of this paper is summarized as follows:

i. A distributed modular IAQM system using sensors nodes for many AQ parameters, a WSN, and an IoT server is developed. The system’s hardware and software are described in detail.
ii. Gateways that ensure transmission from the sensors nodes to IoT servers without data loss are developed. Indeed, the solution includes a mechanism of transmission error detection and packet resubmission in the case of temporary interruption of communication.

The remainder of the paper is organized as follows. Section 2 provides an overall description of the design of the proposed IAQM system. Sample results of data collected with the proposed system are presented and discussed in Section 3. The paper is concluded in Section 4.

## 2. Proposed Indoor Air Quality Monitoring System

**Figure 1:**
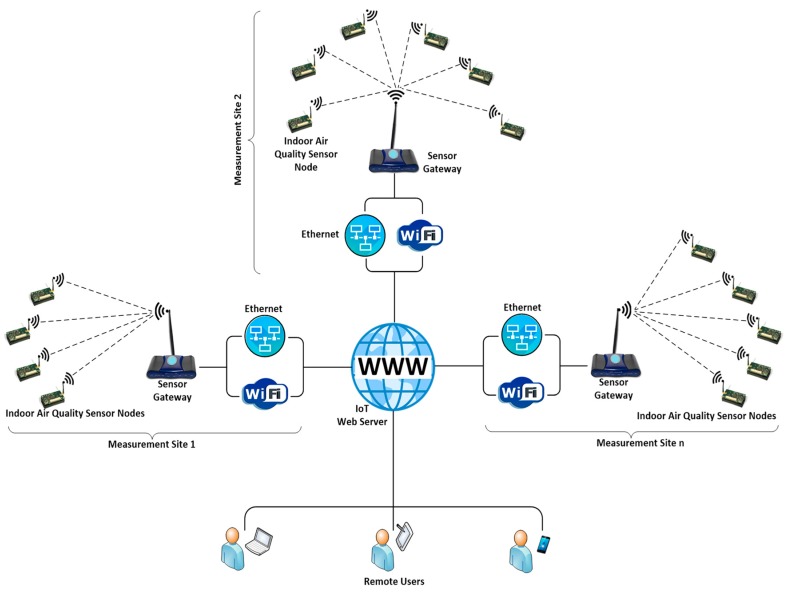
IAQM system architecture.

The overall architecture of the proposed IAQM system is shown in Figure 1. The system can collect AQ data from several sites simultaneously. The collected data are processed and transmitted to an IoT server to be made available to remote users both in graphical and tabular forms. In each measurement site (e.g., a house), several sensor nodes collect IAQ data and send them to a gateway. The gateway processes the received data and posts the aggregated data with timestamp to the external world over the Internet. This section discusses details of the hardware and firmware design of each part of the proposed system.

### 2.1. System Hardware

The system hardware consists mainly of the sensor nodes and gateways, which are described below. A star-network configuration has been adopted in the present application since, in each measurement site, the sensor nodes are scattered near and around their gateway.

#### 2.1.1. Sensor Nodes

**Figure 2:**
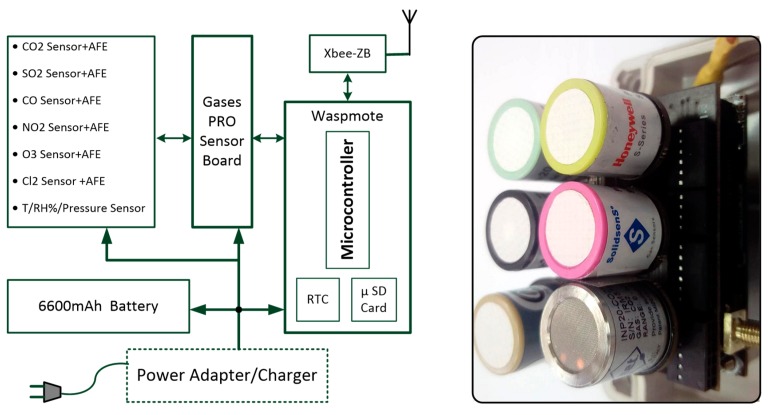
Gas sensors node for IAQM.

Each sensor node (Figure 2) monitors the concentrations of six gases in addition to ambient temperature and relative humidity. The sensor node communicates wirelessly with the gateway through XBee PRO radio modules. A dedicated firmware is developed for the sensor node as described below. Libelium sensor platform was selected for the sensor nodes; this platform is characterized by the modularity of its architecture and by its ability to support several sensors and communication modules. The sensor node includes: (i) a set of calibrated sensors; (ii) a sensors interface board called Gas Pro Sensor Board; (iii) a processing and data storing board, called Waspmote, incorporating an ATmega1281 microcontroller operating at 14.74 MHz, a 128 kB Flash memory, an 8 kB SRAM, a 2 GB SD card, a 32 kHz Real Time Clock (RTC), seven analog inputs, eight digital I/O, two UARTs, one I2C, one SPI, and one USB port; (iv) a Series 2 XBee PRO (one mile line-of-sight range) communication module; and (v) a rechargeable battery with a typical capacity of 6600 mAh. In the present indoor application, the sensor nodes are powered from the mains sockets, and the battery is mainly required for the RTC and backup in the case of temporary power failure.

**Figure 3:**
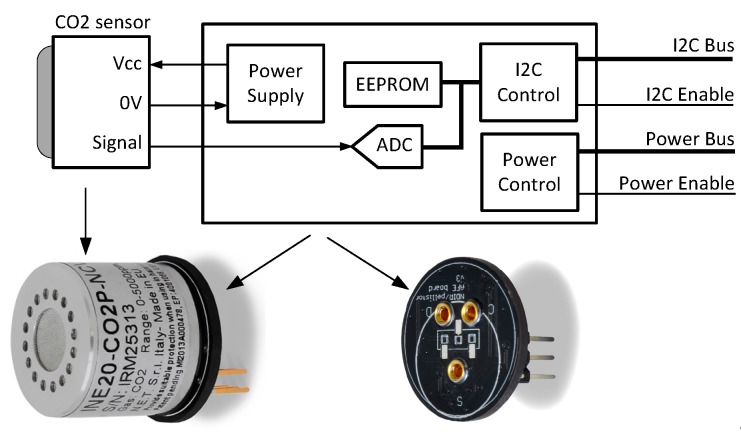
NDIR-type CO_2_ sensor and its interface module.

**Figure 4:**
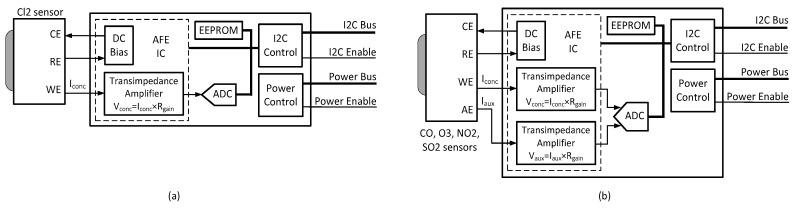
Block diagrams of the interface modules for the: three-electrode (**a**); and four-electrode (**b**) electrochemical sensors.

As shown in Table 1, the sensors included in each node are used for measuring concentrations of CO_2_, CO, SO_2_, NO_2_, O_3_, and Cl_2_; ambient temperature (T); and relative humidity (RH). The CO_2_ sensor is of Non-Dispersive Infrared (NDIR) type and is mounted on a special front-end interface board (Figure 3). By using a suitable infrared source and a compatible light detector, the CO_2_ concentration may be determined by analyzing the optical absorption of the light that passes through the gas. The other gas sensors are of electrochemical type cells operating in amperometric mode (current output), and have either three or four electrodes (Figure 4). A three-electrode device includes a Working Electrode (WE) that reacts with the target gas and generates a current proportional to its concentration, a Counter Electrode (CE) that supplies a current which balances that generated by the WE, and a Reference Electrode (RE) that sets the bias voltage of the WE. The four-electrode device includes an additional Auxiliary Electrode (AE), which is used to compensate the effects of temperature in the baseline current. Each of these electrochemical sensors is mounted on a front-end board, in which the current generated by the WE is converted by a transimpedance amplifier into a voltage for the integrated analog-to-digital converter (ADC). The transimpedance amplifier gain is adjusted by a resistor depending on the range of the target gas concentration; alternatively, an auto-ranging mode may be selected that allows automatic selection of the resistor for maximizing the output voltage to match the ADC full-scale range for accurate conversion. The bias voltage is managed by the analog front-end (AFE) module and by the parameters stored in the EEPROM associated with the sensor. The board for the four-electrode devices includes a second transimpedance amplifier for converting the current of the AE into a voltage. The communication between the sensor interface and other digital devices is done through an I2C bus; which allows measurement data to be available and exchange of commands such as requests to obtain the serial number (stored in the EEPROM) of the sensor or set the gain of the transimpedance amplifier. The sensors are pre-calibrated by Libelium using look-up tables stored in the EEPROM of the front end. Re-calibration of these sensors is done using an in-house developed calibration rig, the details of which will be reported elsewhere. The frequent in-lab calibration of the sensors guarantees the reliability of the collected IAQM data.

The wireless sensor node has to incorporate a suitable module to communicate with the gateway. Sensor nodes deployed inside buildings send data to a gateway with single hop or multi-hop communication modes; the former mode has been chosen in the present work. Power requirement, distance and indoor through-wall communication [16] were the main factors considered when choosing the XBee PRO S2 radio module for the IAQM system. The Waspmote board is fitted with a socket compatible with the chosen XBee PRO S2 radio module, and the gateway described below is also readily compatible with the same module.

#### 2.1.2. Gateway

**Figure 5:**
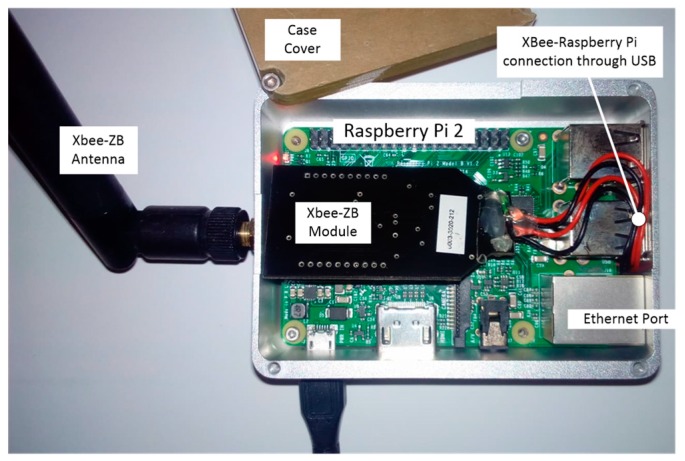
Gateway.

The core of the gateway (Figure 5) is the flexible Raspberry Pi2 model B minicomputer with the following relevant features:

- System on Chip (SoC) Broadcom BCM2835
- 32-bit/700 MHz ARM7 Processor
- 1 GB Memory (SDRAM)
- On-board storage: MicroSDHC slot for up to 32 GB,
- On-board network: 10/100 Mbit/s Ethernet port
- Four USB ports (2.0)
- Power source: 5 V via MicroUSB or GPIO header
- Power rating: 220 mA (1.1 W) average when idle, 820 mA (4.1 W)

The processing power of the Raspberry Pi2 is adequate for the present application. Note that, by using a typical 8 GB micro SD card, the operating system (OS) and the applications consume nearly 2 GB of the memory, and the remaining space (6 GB) is available to store the data measurements. In our system, data are stored every 15 min, and each packet needs 114 Bytes containing node id (2 Bytes), time stamp (10 Bytes), concentrations of six gases (6 × 8 Bytes), temperature (6 Bytes), relative Humidity (6 Bytes), battery level (6 Bytes) and counter (10 Bytes) plus spaces. A gateway with typically five sensor nodes connected to it requires only 114 × 5 × 4 × 24 = 54.72 kB for storing IAQM data in the SD card in .txt format each day. Note that, as described below, a stack is used to temporarily store data in the node in the case of unsuccessful transmission to the gateway; however, the memory space required for this stack is negligible as this is cleared every time temporarily-saved data are re-sent successfully. Therefore, the available memory space of 6 GB is able to store IAQM data for 6,000,000/54.72 = 109,649 days (equivalent to more than 300 years!).

**Figure 6:**
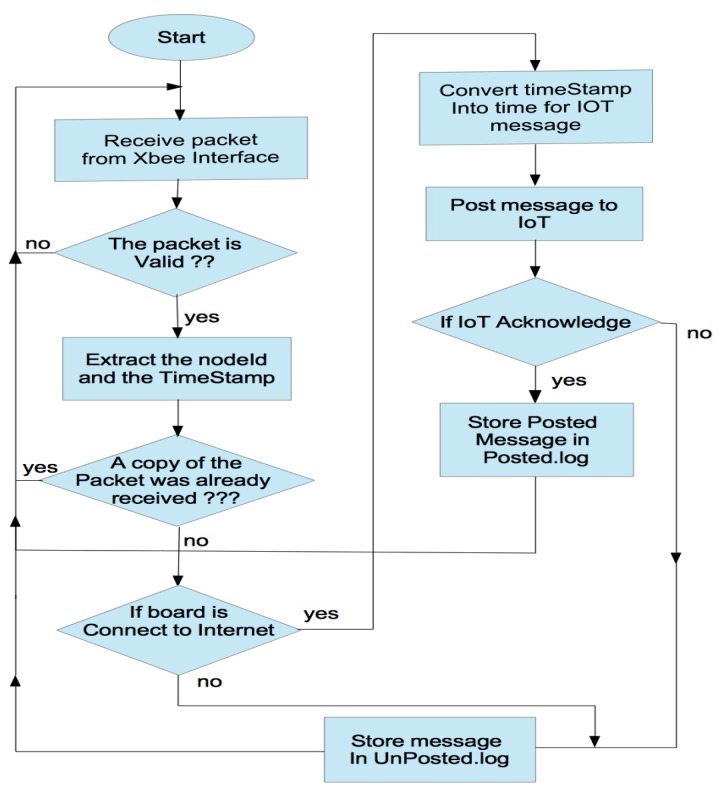
Flowchart of the data processing at the gateway.

The radio module used for communication with the sensor nodes is the XBee PRO S2 (Figure 6). This module communicates with the Raspberry Pi2 via a USB port using a USB-XBee adapter. The Internet connection of the gateway is secured through the Ethernet port of the Raspberry Pi; this can also be done through WiFi using a USB WiFi dongle. Note that Raspberry Pi 3 has built-in WiFi module; therefore, an upgrade from Raspberry Pi2 to Raspberry Pi3 would dispense with the external WiFi module.

### 2.2. System Firmware

The proposed IAQM system includes sensor nodes and gateways, both of which are equipped with communications modules. Each component is managed by a dedicated firmware. The physical layer consists of the radio module (XBee PRO S2). The data link layer consists of the gateway, device manager and communication protocol.

#### 2.2.1. Sensor Node Firmware

On the sensor node, firmware is implemented to operate in one of two modes: (i) calibration mode; or (ii) measurement mode, which is the default mode. As the name implies, the calibration mode is used when calibrating the sensors; this is done in a purpose-built calibration rig that is described in another communication. In brief, the rig consists of a temperature-controlled chamber for housing the sensor node to be calibrated. A computer-controlled gas mixing equipment is used to precisely set the gas composition in the chamber according to a pre-determined schedule. The computer communicates with sensor node to log the sensors readings for each gas-composition set point, and performs polynomial fitting of each sensor output as a function of the true gas concentration (set by a computer-controlled calibration rig). For each sensor, identified by its serial number, a mathematical equation is then determined and stored in the SD card of the sensor node for the correction of the sensor reading. In the measurement mode described in the current work, the processor of the Waspmote board of the sensor node identifies the associated sensors through their serial numbers, which are stored in the EEPROMs of the sensors. To change the operating mode, the sensor node is operated in a server-client architecture where the sensor node is the server. The client node (gateway/PC of the calibration rig) can change the operating mode by sending specific commands to the sensor node. In the measurement mode, the main data acquisition tasks at each sensor node are monitored by the firmware summarized in Algorithm 1. Note that the measurement cycle is set to 15 min.

**Algorithm 1.** Pseudo code of the Firmware at the sensor node operated in the measurement mode1: Call the required libraries (waspXBee and waspSensorPro),2: Initialize and power on each sensor,3: Read serial numbers (stored in the EEPROM of each sensor) of sensors used in the node,4: Wait 90 s for sensors to warm up,5: Acquire 5 readings from each sensor and compute their average,6: Apply the calibration/correction formulas (one for each sensor identified by its serial number) for the sensor reading, 7: Save the timestamped-measured data in SD card,8: Send the data via XBee interface to the gateway,9: Switch the sensors and radio module to sleep mode for 13 min,10: Wake up the sensors and radio module,11: Go to line 4

In addition, at each sensor node in the measurement mode, a mechanism of packet resubmission is triggered when the connection is restored. The gateway always, assigns an absolute counter/index associated to the received packets to identify, which one is missing. The indexes of the missed packets are stored in a specific stack at the gateway. The backward ZigBee communication from the gateway to the nodes is established to send a request to the specific sensor node for the resubmission of the missed packet stored therein. Since the index of each packet is incremented by the sensor node, the acquisition process running at the gateway easily detects the missed index. Algorithm 2 presents a pseudo code of the firmware running during the idle time of the senor board. This firmware ensures the resubmission of the missed packets by checking the size of the stack at the SD card. Once the message is transmitted to the gateway, it will be deleted from the Stack.

**Algorithm 2.** Pseudo code at the sensor node for resubmission of unsuccessfully transmitted packets1: Call the required libraries, waspXBee, waspSensorPro2: While (time < 13 min and Stack is not empty) do3:   Read message from the Stack (SD card)4:   Send the message to the gateway5:   If no acknowledgement received or timeout then6:      Go to line 37:   Else 8:      Remove message from Stack9:   End if10: End while

#### 2.2.2. Radio Module Firmware

A firmware is uploaded onto the radio communication module of the node and the gateway (XBee PRO). This module can be programed as Coordinator, Router or End Device. The coordinator is the radio module responsible of the forming of the network. The coordinator has to manage the security of the network and it selects the appropriate channel, PAN ID, and extended network address. The routers are responsible for routing data between different nodes, and receiving and storing messages intended for their dependent nodes. The routers also are responsible of allowing new nodes to join the network. The End Device is the simple node of the network and it is not allowed to route data. In ZigBee network, only End Device is allowed to switch to sleep mode.

*AT mode (or Transparent mode):* With this mode, all data are sent to the XBee module as in serial transmission (baud rate, parity bit, etc.), without error control. The physical Destination Address is pre-stored in the memory of the radio module. The data are simply transmitted in asynchronous mode. Only serial data transmission is allowed—there is no acknowledgments or transmission requests.

*API mode (Application Programming Interface):* In this mode, data must be formatted in frames with Destination information, and payload. The API mode provides more facilities for the application running on the host to interact with the network. This mode is usually preferred over AT mode since: (1) it allows the transmission of the data to multiple destinations without using command mode; (2) it enables to receive acknowledgment of transmission status for each attempted transmission; and (3) it reduces the power consumption of the network by providing “sleep mode” for the End Devices until data are requested.

In our application, the XBee PRO module at the gateway is configured as coordinator in API mode. However, the XBee PRO module on each sensor is configured as End Device. For each network, a PAN ID value is set for the entire network by the ZigBee Coordinator (ZC) when the Personal Area Network (PAN) is formed and must not change while the PAN is operating. For example, for broadcast transmission, all nodes with the same PAN ID receive the packets but no acknowledgment is sent back. The radio module parameters setup and the upload of the firmware are mainly done using XCTU which is a software tool for the configuration of most RF modules made by Digi International^®^, including the module used in the present work [17]. XCTU uses a serial link to upload customized firmware on the radio modules. In our application, the following main parameters need to be setup using XCTU:

- The PAN ID, which is an ID common to the nodes belonging to the same network. In our application, we configured the Radio module firmware of several End Devices with same PAN ID; the gateway radio module has been configured as coordinator with the same PANID as the End Devices.
- Select the latest firmware for AT or API modes and type of node (Coordinator, Router or End Device).
- The destination address high (DH).
- The destination address low (DL).
- The serial interface parameters: baud rate (BD), Parity, (NB), Stop Bits (SB) to be compatible with board on which the radio module is plugged. For the Libelium Waspmote board used in this work: BD = 115,200.
- Several other standard parameters are set to their default values.

It is important to note that for the API mode on the gateway, Digi International^®^ provides a semi-complete implementation of the XBee PRO binary application programming interface protocol and allows developer to send and receive data without dealing with raw communication details. In our case, the applications on the gateway are based on python codes; hence we installed the python-xbee binary library on the gateway.

#### 2.2.3. Gateway Firmware

The operating system controlling the Raspberry Pi2 is a modified version of Debian Linux, optimized for the Advanced RISC Machines (ARM) architecture. The raspi-config command is used to configure the operating system. The desktop graphic user interface (GUI) on Raspberry Pi is disabled and the secure socket shell (ssh) server is configured through the raspi-config command for remote access to the gateway via Ethernet or WiFi link.

**Figure 7:**
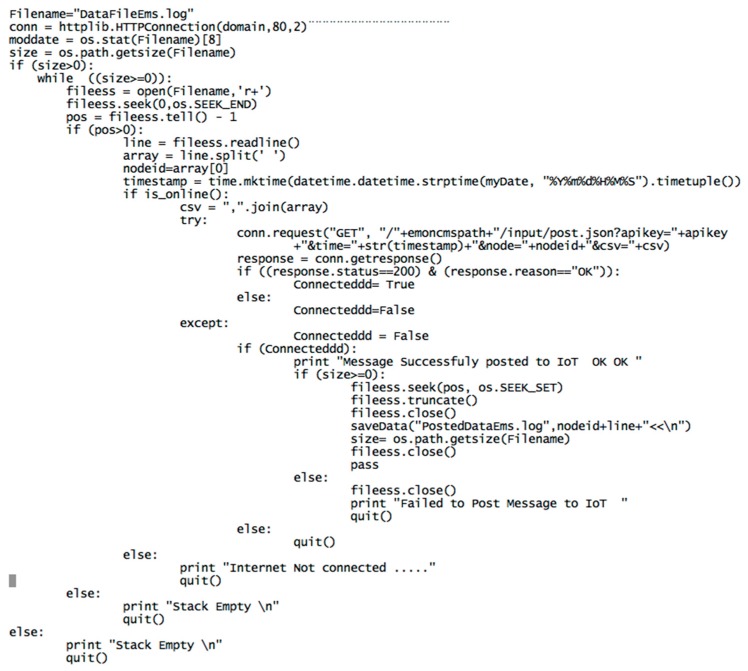
Message resubmission after Internet connection code Fragment.

The main goal of the proposed IAQM system is to provide a reliable mechanism to collect IAQ data and post them to an IoT server for remote users. On the gateway, two main applications are implemented. The first one supports the process that reads the data from the radio interface and the protocols to manage the message exchanges between the gateway and the remote IoT server via Internet link. The second application serves as rescue for the restoration of packets, which are lost in the communication channels. The flowchart of the first application is illustrated in Figure 6. The python script ensuring the reposting of the messages missed by the IoT server is given in Figure 7. Once received through the radio link, the data are processed and stored locally at the gateway for further use or backup. Prior to storing data locally and transmitting them to the IoT server, the data are processed to extract the fields from the received packets using the XBee library.

To ensure the integrity of the data packets when Internet connection is lost, the gateway detects the connection failure, stores temporarily un-transmitted packets in a stack and then posts these packets using past timestamps once the Internet connection is restored again. Note that if only 0.01% of the data storage space of the gateway SD card is reserved for the stack, data can be restored even in the event of a very long Internet interruption. To illustrate this, consider a gateway fitted with an SD card of 8 GB storage capacity, 6 GB of which are used for IAQ data storage, and communicating with typically five sensor nodes; this arrangement requires 54.72 kB for storing IAQM data in the SD card in each day (see Section 2.1.2). Under these conditions, the stack can store data during0.01% × 6 GB/54.72 kB/day ≈ 11 days of Internet disconnection.

#### 2.2.4. Posting Data on the IoT Server

The IoT application used to post the collected data is the Emoncms, which is a powerful open-source web-app developed originally for processing, logging and visualizing energy-related data. Emoncms provides a complete hardware and software solution available for users. The software is very specific to the marketed hardware developed for the project openenergymonitor.org. Users of Emoncms are invited to create an account and obtain a private space for their data. For each new account, the user has to create a login and a password, and the system generates two ApiKeys, one for simply reading and the other for writing. The web-application enables the user to setup the inputs, feeds, graph, dashboard, the user account and profile. Some APIs are provided by Emoncms for data input posting and feeds posting.

One of the contributions in this work is the development of specific and personalized software suited for the IAQM project without the need for the specific Emoncms hardware. This is achieved by adapting the gateway (standard Raspberry Pi2) to communicate directly with Emoncms application. Simply using the standard API provided by Emoncms and the write ApiKey related to the created account does posting the packets of IAQM data directly from the gateway to Emoncms.

Usually the gateway communicates with the IoT application using a specific protocol suited for devices with limited resources such as battery capacity, low memory, reduced processing capability and vulnerable radio conditions. Hence, standard TCP/IP protocol stack is not useful for IoT devices. In the new protocols, developed for IoT applications, the addressing, such as in IPv6, is considered since in modern applications the number of nodes has greatly increased. New standard for IoT specific protocols was developed: IPv6 over low-power wireless personal area networks (6LoWPAN).

At the application layer, hypertext transfer protocol (HTTP) is the well-known way to retrieve and request data via the web architecture. Embedded devices are, in contrast, based on new protocol named representation state transfer (RESTful), which is compatible with HTTP for devices with limited resources. For the application layer, the Internet Engineering Task Force (IETF) group has developed a specified application layer protocol for the IoT, called constrained application protocol (CoAP). Message queue telemetry transport (MQTT) is a protocol layer for messaging standardized in 2003 [18], and aims to connect embedded devices with applications and middleware.

**Figure 8:**
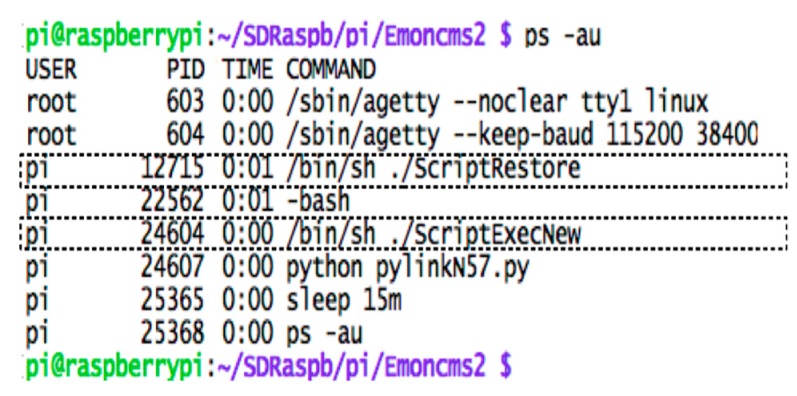
The main scripts running in the gateway.

**Figure 9:**
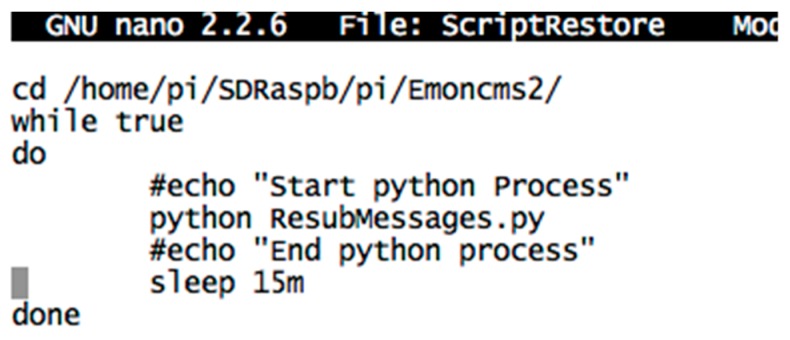
Shell script managing the periodic execution of the message resubmissions.

Data is posted from the gateway to the IoT platform using the specific APIs and the required APIkeys. The main processes running continuously in the gateway are ScriptExecNew and ScriptRestore as illustrated in the screenshot shown in Figure 8. ScriptExecNew calls a python script, which manages the real-time acquisition of the messages from the radio interfaces and the analysis and the processing of these messages before posting them to the remote IoT. In the posting script, the posting API provided by Emoncms returns a response of the result of the post. The process checks whether data have been successfully received at the IoT platform: if {(response.status = 200) and (response.reason = ”OK”)} then the IoT web-server has successfully received the packet otherwise the packet is not posted either because the connection is not established or because the write APIKey is not correct. In that event, the unposted packet is added to a stack. Another process, running in parallel, is idle during 15 min, and then wakes up to check if the stack is not empty, and then if the connection is restored. A python script running in the gateway manages the mechanism of resubmitting messages after Internet disconnection. This script, uses as entry, the stack of un-transmitted messages. This stack consists of list of messages stored in DataFilesEms.log. As may be seen in the code fragment in Figure 7, a first test of the size of DataFilesEms.log ensures the control of the script. Starting from the end, the last message is posted to the IoT using its timestamp. This enables the IoT to place the packet in a past and right position, identified by the timestamp, at the IoT database. The successfully posted message is then deleted from the stack using the command fileess.truncate(). The process of posting ends when the stack is empty. This process runs again after fixed time duration of 15 min according to the shell script shown in Figure 9.

## 3. Results

In this section, we will focus on the evaluation of the performances of the proposed IAQM system. The performance of the communication using the XBee PRO radio modules is evaluated indoor in a large building at Qatar University, Doha, Qatar. To demonstrate the operation of the proposed IAQM system, sample indoor air quality data are collected over an extended period in two remote locations at Qatar University. Comprehensive assessment of indoor air quality in various locations in Doha will be reported in a dedicated future communication.

### 3.1. Evaluation of the Packet Loss Rate in an Indoor Environment

**Figure 10:**
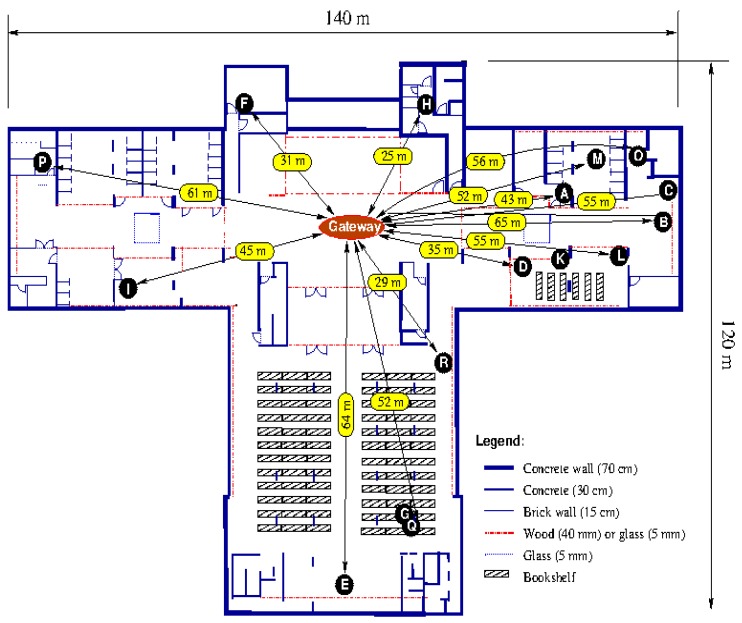
Floor plan of Qatar University library building used for evaluating packet loss of the radio transmission channel.

To evaluate the communication between the sensor nodes and the gateway using the XBee PRO radio modules, the packet loss rate was evaluated inside one of the floors of the main Library building at Qatar University. The selected building is large and includes various obstacles (e.g., glass, concrete and brick walls, large wooden doors and metal book shelves). Figure 10 shows the floor plan layout of the floor used in these tests. The gateway was installed in a central location, 1.5 m from the floor, in the building as shown in the figure. The sensor node was then positioned, 1.5 m from the floor, at various locations labelled A to R in Figure 10; this allowed assessment of data loss under different scenarios (the End Device and the gateway can be separated by glass, concrete or brick walls, and various combinations of these). The transmitting sensor node was configured as End Device and the gateway as Coordinator; both operated in the API mode. From each sensor node location, 5000 data packets (each made up of 114 Bytes) have been transmitted to the gateway, in both unicast and broadcast transmission modes separately. Assessment of the number of packets successfully received by the gateway enables determination of the packet loss probability in each transmission mode for each node location. For the broadcast transmission, the End Device sends three successive packets and the receiver do not transmit any acknowledgment or transmission status. However, for the unicast mode, the End Device sends one packet and waits for acknowledgment. If the acknowledgment is negative, it resends the packet until successful transmission or elapsed fixed time out of 5 s, which stops the transmission and considers that packet is lost.

**Figure 11:**
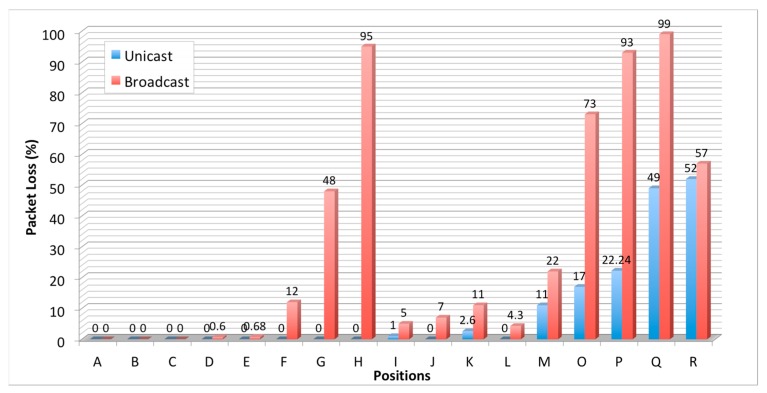
Percentage of packet loss for the various locations of the radio transmitter (shown in Figure 10).

The results in Figure 11 show that the obstacles and their numbers are the main factors affecting transmission hence deliverability, and that the unicast transmission is superior to the broadcast counterpart. In addition, these results show that transmission through the double and triple obstacles is strongly attenuated and the packet loss probability is very high particularly for broadcast transmission. In cases of double and triple concrete wall obstacles (as in positions M, O, P and Q), packet loss of unicast transmission is at least 50% lower than that of broadcast transmission. For single concrete walls (as in positions H, I, J, K, and L), the unicast transmission remains very reliable, with packet loss probability close to zero; however, for the broadcast transmission, the packet loss is very high. For simple and double glass wall obstacles (as in positions A, B, C, D and E), the packet loss probabilities for both unicast and broadcast transmission are close to zero.

### 3.2. IAQM at Qatar University

**Figure 12:**
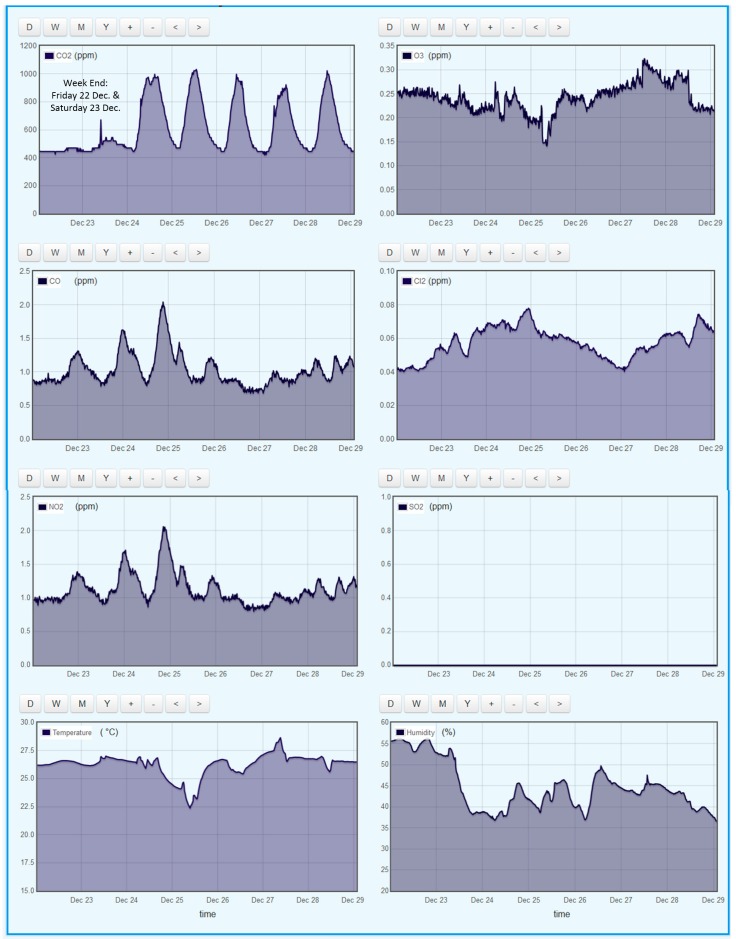
Indoor air quality data (as displayed by the online Emoncms IoT) collected in an office space at Qatar University during December 2017.

Sample IAQM results have been obtained in Qatar University campus using the proposed system. The chosen locations are: (i) a large open-space office in the research building of the college of engineering; and (ii) a chemical engineering research lab. Full-time researchers who report to work from 7:30 a.m. to 3:15 p.m., five days per week, use the office space. This office space is ventilated through a central air conditioning system. The laboratory is used by a limited number of researchers, and is accessible by authorized users through RFID-controlled doors; this lab is equipped with fume-hoods and is ventilated through the central air-conditioning system. Figure 12 gives a screenshot of the office space results as displayed by the online IoT Emoncms of the proposed system. The Emoncms allows the user to select a time window of IAQM results up to one year; the results shown are for one week (starting 22 December 2017). One may notice that, during working days, the CO_2_ concentration starts rising at 7:00 a.m. and begins to fall around 4:00 p.m.; the concentration peaks around 1000 ppm, which is the recommended limit for eight-hour exposure according to WHO and ASHRAE guidelines. During the weekend days (Friday and Saturday), the CO_2_ concentrations are very low, explained by the absence of activities in the office, except for the cleaning activity on Saturday, which results in a slight increase in CO_2_ levels in the building. The O_3_ levels are around 0.2 ppm and appear to be five times higher than the recommended eight-hour exposure limit (0.05 ppm) set by WHO; nevertheless the 0.2 ppm is within the sensor’s accuracy (see Table 1) and hence it is recommended to change the O_3_ sensor with a more accurate and a lower measurement range one. The levels of CO (around 1 ppm) are well below the WHO-recommended eight-hour exposure limit of 9 ppm; however, the 1 ppm accuracy of the sensor does not enable accurate measurement of the CO concentration in the range encountered in our application. The Cl_2_ levels are around 0.05 ppm; these are slightly above the WHO limit of 0.034 ppm although the widely accepted 15-min exposure limit is 0.5 ppm [19,20]. The NO_2_ levels appear to be around ten times higher than the one-hour exposure limit of 0.1 ppm set by the US Environmental Protection Agency (EPA) [21]. Note that, given that NO_2_ sensor’s accuracy of 0.1 ppm, this does not seem adequate for checking compliance with EPA limit. It is important to note that the CO and NO_2_ levels show high correlation [22]; these pollutants are likely generated by the nearby traffic and by the gas processing facilities located north of Doha. The results of SO_2_ measurements indicate traces, which cannot be detected by the sensor used; the accuracy of this sensor is 0.1 ppm. In the US, various states recommend 3-h exposure limit of 0.5 ppm [23]. The Cl_2_ levels stayed around 0.05 ppm in the office space.

**Figure 13:**
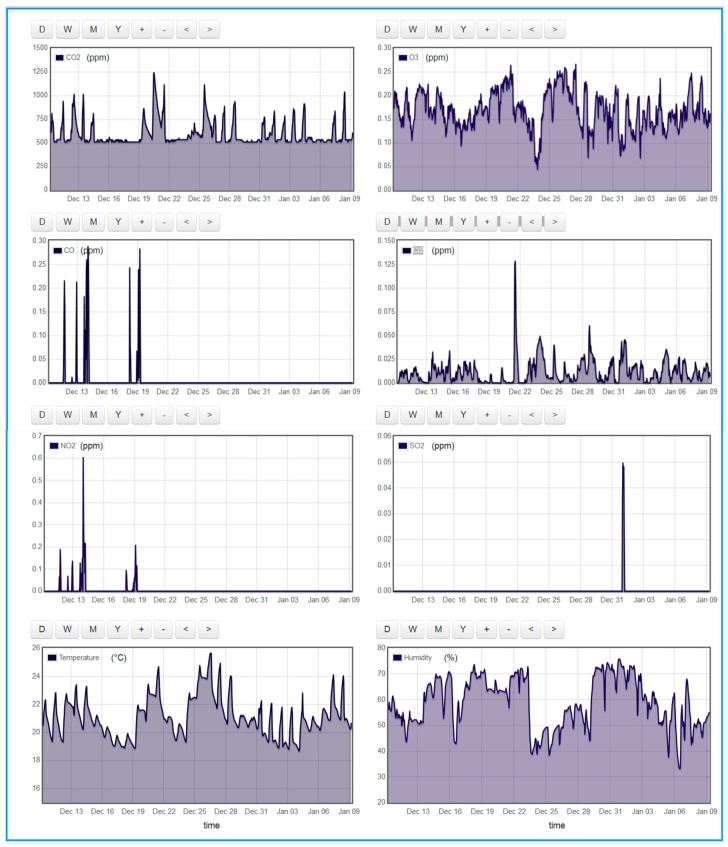
Emoncms IoT-displayed indoor air quality data collected in a Chemical Engineering research lab of Qatar University (9 December 2017 to 9 January 2018).

Figure 13 depicts IAQM results obtained in a chemical engineering lab during the period 9 December 2017 to 9 January 2018. In this lab, the students manipulate experiments involving several gases such CO_2_, Cl_2_, and CO. From the CO_2_ results, we noticed that CO_2_ concentration is mainly dependent on the presence of students and on the time when their experiments release CO_2_ gas. The results depict sensing of occasional traces of CO, CL_2_, NO_2_ and SO_2_ released by some of the experiments carried out in the laboratory. In addition, as we interpreted before, we notice that CO and NO_2_ levels are correlated.

## 4. Conclusions

This work presented a modular end-to-end indoor air quality monitoring (IAQM) system made up of a wireless sensors network, gateways and an IoT server. The system enables monitoring of six gases in addition to temperature and humidity at different sites simultaneously. The reliability of radio communication between sensing nodes and gateways, and the Internet communication reliability between the gateways and servers has been particularly addressed. This is implemented by a backup and data resubmission mechanism in the case of radio communication failure or Internet disconnection. Additionally, the modularity of the system allows scalability and hence the system may integrate a large or limited number of sensors nodes to suit different applications. The design details of the hardware and software components of the end-to-end system have been described. In addition, the collected data were thoroughly discussed and recommendations were provided.

The developed platform will be used to collect IAQ data in various locations in Doha-QATAR, and over an extended period covering different seasons. The same platform can also be easily modified to assess air quality in relation to gases (such as VOC) other than those described in this work. The choice of IAQ parameters depends on the environment in which the study is performed since the sources of pollution differ from one place to another.

## Figures and Tables

**Table 1:** Specifications of the sensors used.

IAQ Parameter	Sensor Model	Nominal Range	Accuracy (ppm)	Response Time * (s)	Sensor Type
SO_2_	4-SO2-20	0–20 (ppm)	±0.1	T90 ≤ 45	Electrochemical
NO_2_	4-NO2-20	0–20 (ppm)	±0.1	T90 ≤ 30	Electrochemical
O_3_	OX-A431	0–18 (ppm)	±0.2	T90 ≤ 45	Electrochemical
CO_2_	INE20-CO2P-NCVSP	0–5000 (ppm)	+/−50 (0–2500 ppm) +/−200 (2500–5000 ppm)	T90 ≤ 60	NDIR
CO	4-CO-500	0–1000 (ppm)	±1	T90 ≤ 30	Electrochemical
Cl_2_	4-Cl2-50	0–50 (ppm)	±0.1	T90 ≤ 30	Electrochemical
T & RH	BME280	−40–85 °C (T) 0–100% (RH)	±1 °C (T) ±3% (RH)	T63 ≤ 1.65 (T) T63 ≤ 1 (RH)	-

(*) T90: rise time to 90% of final value; T63: rise time to 63% of final value.
